# An unusual outbreak in the Netherlands: community-onset impetigo caused by a meticillin-resistant *Staphylococcus aureus* with additional resistance to fusidic acid, June 2018 to January 2020

**DOI:** 10.2807/1560-7917.ES.2022.27.49.2200245

**Published:** 2022-12-08

**Authors:** Karuna E.W. Vendrik, Ed J. Kuijper, Marieke Dimmendaal, Welmoed Silvis, Els Denie-Verhaegh, Annemarie de Boer, Bent Postma, Annelot F. Schoffelen, Wilhelmina L.M. Ruijs, Fleur M.H.P.A. Koene, Mariska Petrignani, Mariëtte Hooiveld, Sandra Witteveen, Leo M. Schouls, Daan W. Notermans, MRSA consortium, MRSA consortium, Robert F.W. Schuurman, Bart Schipper, Margreeth J.C. Oijevaar, Lennard F.M. Hiltermann, Geert-Jan M. van Loenen, Renate Jurgens, Wieke I. Noordenbos, Gert-Jan Leeflang, Gertrude Janssen, Bas B. Wintermans, Maurine A. Leversteijn-van Hall, Wouter van den Bijllaardt, Rosa van Mansfeld, Karin van Dijk, Bas Zwart, Bram M.W. Diederen, Andreas Voss, Julia W. Dorigo-Zetsma, Alewijn Ott, Joke H. Oudbier, Marleen van der Vusse, Anneloes L.M. Vlek, Anton G.M. Buiting, Lonneke Bode, Sunita Paltansing, Arjanne J. van Griethuysen, Martijn den Reijer, Marijke J.C.A. van Trijp, Nathalie D. van Burgel, Anouk E. Muller, Michael P.M. van der Linden, Michiel van Rijn, Maurice J.H.M. Wolfhagen, Karola Waar, Eva Kolwijck, Tanja Schulin, Marjolein Damen, Sander Dinant, Suzan P. van Mens, Damian C. Melles, James W.T. Cohen Stuart, Paul Gruteke, Ilse T.M.A. Overdevest, Alje P. van Dam, Ianthe Maat, Boulos Maraha, Jan C. Sinnige, Eva Mattsson, Ellen M. Mascini, Arjen Stam, Eefje de Jong, Saara J. Vainio, Esther Heikens, Radijn Steingrover, Annette Troelstra, Eric Bathoorn, Thera A.M. Trienekens, Dick W. van Dam, Els I.G.B. de Brauwer, Frans S. Stals.

**Affiliations:** 1 National Institute for Public Health and the Environment (Rijksinstituut voor Volksgezondheid en Milieu, RIVM), Bilthoven, the Netherlands; 2 Leiden University Medical Center, Leiden, the Netherlands; 3 Municipal health service North and East Gelderland, Warnsveld, The Netherlands; 4 Laboratory for Medical Microbiology and Public Health (LabMicTA), Hengelo, The Netherlands; 5 Slingeland Hospital, Doetinchem, The Netherlands; 6 Praktijk voor huisartsenzorg Whemerhof, Dinxperlo, The Netherlands; 7 Public Health Service of Amsterdam, Amsterdam, The Netherlands; 8 Medical Laboratory Services, Willemstad, Curacao; 9 Nivel, Utrecht, The Netherlands; 10 The members of the MRSA consortium are listed under Collaborators

**Keywords:** MRSA, exfoliative toxins, fusic acid resistance, fusC, Outbreaks, Community Acquired Infections

## Abstract

In this retrospective observational study, we analysed a community outbreak of impetigo with meticillin-resistant *Staphylococcus aureus* (MRSA), with additional resistance to fusidic acid (first-line treatment). The outbreak occurred between June 2018 and January 2020 in the eastern part of the Netherlands with an epidemiological link to three cases from the north-western part. Forty nine impetigo cases and eight carrier cases were identified, including 47 children. All but one impetigo case had community-onset of symptoms. Pharmacy prescription data for topical mupirocin and fusidic acid and GP questionnaires suggested an underestimated outbreak size. The 57 outbreak isolates were identified by the Dutch MRSA surveillance as MLVA-type MT4627 and sequence type 121, previously reported only once in 2014. Next-generation sequencing revealed they contained a fusidic acid resistance gene, exfoliative toxin genes and an epidermal cell differentiation inhibitor gene. Whole-genome multilocus sequence typing revealed genetic clustering of all 19 sequenced isolates from the outbreak region and isolates from the three north-western cases. The allelic distances between these Dutch isolates and international isolates were high. This outbreak shows the appearance of community-onset MRSA strains with additional drug resistance and virulence factors in a country with a low prevalence of antimicrobial resistance.

**Key public health message**

**What did you want to address in this study?**

A large regional community outbreak of impetigo, mainly involving young children, was recognised when several general practitioners reported unresponsiveness to antibiotic treatment. Meticillin-resistant *Staphylococcus aureus* was cultured and found resistant to other antibiotics used to treat impetigo. We estimated the outbreak size and described which interventions resulted in a rapid decrease of new cases.

**What have we learnt from this study?**

In a country with a low prevalence of antibiotic resistance, outbreaks with new MRSA types containing additional resistance genes and virulence factors can occur and spread in the community, especially amongst young children. MRSA community outbreaks are difficult to recognise, but monitoring the prescription data of pharmacies can provide information on the start and extent of an outbreak.

**What are the implications of your findings for public health?**

National guidelines for general practitioners should include advice for better microbiological diagnostics for skin infections that do not respond to first-line antibiotic treatment. Furthermore, prescription data from pharmacies can be useful to assess the extent of an outbreak.

## Background

Meticillin-resistant *Staphylococcus aureus* (MRSA) is a well-known nosocomially transmitted pathogen [1]. However, community-onset MRSA (CO-MRSA) is also regularly reported, frequently causing skin- and soft tissue infections among young children [2,3]. In contrast to hospital-onset MRSA, CO-MRSA strains typically have increased virulence and fitness [4]. CO-MRSA strains frequently carry Panton-Valentine leukocidin (PVL) encoding genes, possibly associated with development of severe, purulent skin and soft tissue infections and necrotising pneumonia [1]. Additionally, other virulence genes can be present, such as exfoliative toxin genes [5]. Exfoliative toxins can lead to the staphylococcal scalded skin syndrome (SSSS), most commonly observed in neonates and children below 5 years of age [6]. In older children and adults, expression of these genes can lead to a milder form of SSSS, bullous impetigo [7].

Impetigo is a contagious superficial infection of the skin, mostly caused by *S. aureus*. It is mainly observed in children and often presents as facial lesions. It spreads through direct skin-to-skin-contact or indirectly via touched items. In the Netherlands, as in other European countries, impetigo is treated primarily with topical fusidic acid [8].

The Netherlands has a low MRSA prevalence with less than 1% of all invasive isolates being resistant to meticillin in 2019 [9]. Since 1989, the National Institute for Public Health and the Environment (RIVM) has received MRSA isolates from diagnostic laboratories, derived from routinely submitted patient samples, for analysis. Clinical and epidemiological data, extracted from electronic patient medical records, are collected through the Type-Ned MRSA digital exchange system, from all but one (n = 55) Dutch medical microbiology laboratory. This system is frequently used to study MRSA transmission and was the basis for the recognition of a community outbreak with an MRSA sequence type (ST) 121 strain.

## Outbreak detection

In July 2019, several general practitioners (GPs) in the eastern part of the Netherlands noticed a rapidly increasing number of impetigo cases unresponsive to topical fusidic acid treatment. Cultures showed the samples contained MRSA and they were sent to the RIVM as part of the national MRSA surveillance. Typing showed the MRSA isolates were of multilocus variable-number tandem repeat analysis (MLVA) type MT4627. This MLVA-type had been found in only three earlier samples from the MRSA surveillance, one from 2014 and two from 2018. The two 2018 isolates were deemed part of this outbreak, since the first of these two isolates was also from the eastern part of the Netherlands. A study group was initiated, composed of general practitioners, clinical microbiologists, epidemiologists, public health infectious disease specialists and pharmacists to assess the size of the possible outbreak and provide recommendations to prevent further spread. This report shows the results of the retrospective outbreak investigation with an analysis of its extent and clinical, microbiological and genomic characteristics.

## Methods

### Case definition and other definitions

A case was defined as a person infected or colonised with MRSA MLVA-type MT4627 in the period from June 2018 to January 2020, identified through the Dutch MRSA surveillance programme. Only one isolate per person was included.

**Figure 1:**
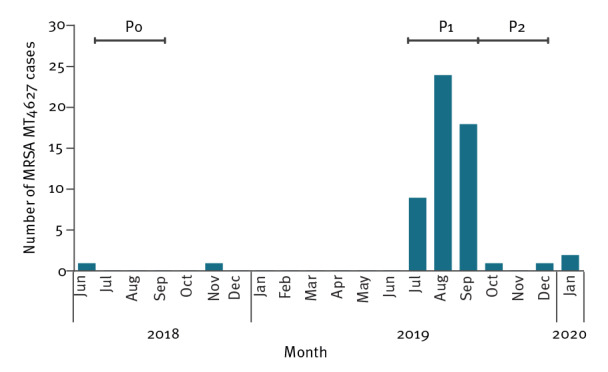
Meticillin-resistant *Staphylococcus aureus* multilocus variable-number tandem repeat analysis type MT4627 cases by sample month, the Netherlands, June 2018 to January 2020

Three different time periods were defined: the peak outbreak period (P1) with more than three MRSA MT4627 isolates detected per month; the 3 months thereafter (P2); and the same calendar period as P1, in the previous year (P0) (Figure 1).

MRSA: Meticillin-resistant *Staphylococcus aureus*.

P0: the same calendar period as the peak outbreak period in the year before the peak outbreak period; P1: peak outbreak period; P2: the 3 months after the peak outbreak period.

Data source: Type-Ned database.

The outbreak region was defined as the municipal health service region Noord-Oost Gelderland, in the eastern part of the Netherlands with 22 municipalities, 827,731 inhabitants [10] and 261 GPs. The cases were from eight villages/towns (16 GPs) in five municipalities. We defined an adjacent region, municipal health service region Twente, located adjacent to the outbreak region with 14 municipalities, 631,064 inhabitants [10] and 195 GPs. The municipal health service region Friesland was selected as the control region, located in the north of the Netherlands with 18 municipalities, 649,957 inhabitants [10] and 198 GPs. The control region was only used for analysing data from the Netherlands Institute for Health Services Research (Nivel) Primary Care Database (see below) [11].

### Epidemiological and clinical characteristics

Epidemiological and clinical characteristics were provided retrospectively by GPs, the municipal health service and two regional medical microbiology laboratories from the outbreak region. Furthermore, clinical and epidemiological data from MRSA MT4627 cases were extracted from the Type-Ned surveillance database.

Severe impetigo was defined as SSSS, severe generalised bullous impetigo, hospital admission and/or death due to impetigo with MRSA MT4627.

### Data from general practices and prescriptions of mupirocin and fusidic acid

To obtain insight into the extent of the outbreak, GPs from the outbreak region (n = 5 practices) and from the adjacent region (n = 11) were requested to fill out a questionnaire on the number and characteristics of impetigo cases, diagnostics and treatment regimens (Supplementary Table S1). The GPs from the outbreak region were selected by the municipal health service based on the highest number of cases. The GPs from the adjacent region were selected as a control by a regional clinical microbiologist. Furthermore, weekly data from electronic medical records of sentinel general practices participating in the Nivel Primary Care Database [11] were obtained and analysed. Nivel is an independent foundation. The Nivel Primary Care Database uses routinely recorded data from healthcare providers to monitor health and utilisation of health services in a representative sample of the Dutch population.

Data on the number of prescriptions for mupirocin and fusidic acid for P0, P1 and P2 were requested from three pharmacists in the outbreak region and five in the adjacent region. Pharmacies that provided medication for patients from the GPs that filled in the questionnaire were selected.

### Microbiological and genomic analysis

Routine susceptibility data from the MRSA MT4627 isolates according to the European Committee on Antimicrobial Susceptibility Testing (EUCAST) breakpoints were provided by two regional laboratories [12]. MLVA was performed as described previously [13]. Next generation sequencing (NGS) was performed using the Illumina HiSeq 2500 Sequencing System (BaseClear B.V., Leiden, the Netherlands). Genetic relatedness between isolates was assessed using the NGS data in the COL-based whole-genome multilocus sequence typing (wgMLST) scheme [14] available via SeqSphere software version 6.0.2 (Ridom GmbH, Münster, Germany). All NGS-derived data were imported into BioNumerics version 7.6.3 (Applied Maths, Sint-Martens-Latem, Belgium) for analysis. In the comparison of isolates using wgMLST, missing genes are ignored to prevent for unrealistic genetic distances of isolates as in core-genome MLST. The wgMLST results were visualised with a minimum spanning tree using BioNumerics. A genetic cluster was defined as two or more isolates with an allelic distance of 15 or less and with similar virulence and antimicrobial resistance (AMR) genes and plasmid replicons. Furthermore, the genomes from the sequenced isolates were compared with genomes available in the National Center for Biotechnology Information (NCBI) and Sequence Read Archive (SRA) database. AMR genes were identified via ResFinder software [15] and only AMR genes with > 99% sequence identity and sequence coverage with the reference sequences were included. For the identification of virulence factors, VirulenceFinder was used with a cut-off of > 90% identity and coverage. The presence of plasmid replicons was assessed using PlasmidFinder software [16] with a 100% identity and coverage cut-off.

Raw NGS sequence data of all sequenced isolates were deposited in the Sequence Reads Archive of NCBI under BioProject ID PRJNA807127.

### Data presentation

Data are presented as n (of total n) for categorical variables and, for numerical variables that have a skewed distribution, median (interquartile range, IQR). Using Nivel data, the cumulative number of weekly impetigo consultations per 10,000 persons was calculated for P0, P1 and P2 for (i) the outbreak region; (ii) the adjacent region; (iii) the whole country except for the outbreak region and (iv) the control region. Age distribution data from Statistics the Netherlands (CBS) [17] were analysed to verify whether the adjacent and control regions had a similar percentage of children aged 0–10 years to the outbreak region.

Prescription data from pharmacies were expressed as number of mupirocin and fusidic acid prescriptions. The Nivel and prescription data were analysed, and the cumulative number of weekly impetigo consultations per 10,000 persons and the number of mupirocin and fusidic acid prescriptions from the P1 period were descriptively compared to data from the P0 and P2 periods. In all analyses, a complete case analysis was performed. The number of persons with available data per variable was mentioned.

The number of undetected outbreak cases was estimated by asking GPs how often samples of clinically diagnosed impetigo cases were not cultured. An adjacent and/or control region was included to examine whether impetigo rates in these regions were also increased during the outbreak period.

## Results

### Epidemiological and clinical characteristics

In the period June 2018 to January 2020, 57 persons were identified with infection or colonisation with MRSA MLVA-type MT4627. Six symptomatic family members who were treated for an infection without culture were not included in this analysis. The epidemiological curve is shown in Figure 1. The period July to September 2019 (n = 51) was considered to be the peak outbreak period (P1).

Of the 57 cases involved in the outbreak, 49 were from five municipalities located in the eastern part of the Netherlands (the outbreak region). These cases had an epidemiological link to three cases found in the north-western part of the Netherlands through family contact. Visiting school, family and friends was considered to be the cause of the spread of the outbreak. The characteristics of the 57 cases with MRSA MT4627 are shown in Table 1. In total, 25 were female and 32 were male. Of these, 47 were children (20 females, 27 males; median age 5 years, IQR: 3–7). The adults (n = 10) were between 28 and 48 years old. Among 41 cases with known family relationships, there were 26 different families involved. Among all 57 cases, 49 had skin lesions caused by MRSA (42 children and seven adults). None of the cases with MRSA MT4627 were admitted to a hospital at the time of sampling, but one impetigo case and one carrier case (positive family member) were outpatients. Only one impetigo case was diagnosed with severe generalised bullous impetigo, for which the patient was later admitted to the hospital. Seven of 39 cases with available information on travel history had travelled to a foreign country. In 2018, the first detected case from the outbreak region had not recently travelled abroad. In 2019, the first detected MRSA MT4627 case and two cases that were family members of this case, had visited Germany in the previous 2 months without hospital admission. Furthermore, two cases from 2019 had recently visited Morocco and two from 2019 and 2020 the Maldives, but hospital admissions were unknown.

**Table 1:** Characteristics of all meticillin-resistant *Staphylococcus aureus* multilocus variable-number tandem repeat analysis type MT4627 outbreak cases in the Netherlands, June 2018 to January 2020 (n = 57)

	n	n total^a^
**Median age in years (IQR)**	6 (4–10)	57
< 18 years old	47	57
**Sex**		
Female	25	57
Male	32	57
**Infection**	49	57
**Infected tissue/organ**		
Skin	49	49
**Location of skin infection**		
Arms and/or fingers	4	23
Buttocks	6	23
Leg	6	23
Abdomen	1	23
Face	5	23
Chest and leg	1	23
**Severity of infection**		
Staphylococcal scalded skin syndrome	0	46
Severe generalised bullous impetigo	1	46
Hospital admission due to MRSA MT4627 infection	1	46
Death due to outcome of MRSA MT4627 infection	0	46
**Material**		
(Swab of) skin	5	57
Pus	20	57
Swab of throat	2	57
Swab of throat and nose	1	57
Swab of throat, nose and perineum	2	57
Swab of nose	7	57
Swab of perineum	1	57
Wound fluid	19	57
**Culture ordered by**		
Hospital	2	57
General practitioner	55	57
**Possible risk factors**		
Long-term care facility resident	0	54
Profession with direct patient care	3	54
Profession with animal contact	0	54
Foreign travel in previous 2 months	7	39
**Comorbidity**		
Diabetes and multiple sclerosis	1	31
No comorbidity	30	31

IQR: inter-quartile range; MRSA: meticillin-resistant *Staphylococcus aureus*.

^a^ With available information.

Data source: Type-Ned database.

Of the 57 cases, 8 were carriers.

### Data from general practices and prescriptions of mupirocin and fusidic acid

Three GPs in the outbreak region and eight in the adjacent region responded to the request for information and filled in a questionnaire (Supplementary Table S1). Similarly, two pharmacists from the outbreak region and two from the adjacent region provided data on fusidic acid and mupirocin prescriptions. An increase in cases with impetigo unresponsive to fusidic acid was observed among all GPs in the outbreak region and adjacent region. The majority of the outbreak region GPs reported that 50–75% of patients did not respond to fusidic acid in the outbreak period, which was 25–50% for adjacent region patients. All three GPs from the outbreak region sent samples for culture in case of no response during the outbreak period, in contrast to none of the eight GPs in the adjacent region. One GP from the outbreak region and five from the adjacent region noticed an atypical clinical picture or atypical locations of impetigo. The GP from the outbreak region mentioned that more bullous impetigo was observed. In case of no response to fusidic acid, two of three GPs in the outbreak region prescribed locally applied mupirocin. All responding GPs from the adjacent region started flucloxacillin or azithromycin orally or clindamycin lotion locally when fusidic acid was not effective.

The data from Nivel are shown in Supplementary Table S2. The cumulative weekly number of impetigo consultations per 10,000 persons in the outbreak region was 77.0 in P1, compared with 59.4 in P0 and 49.0 in P2. Data from patients in the adjacent and control regions with a similar age distribution to the outbreak region and data from the whole country showed slightly lower cumulative numbers of consultations in P1 compared with P0, and a rapid decline in P2. The outbreak region had the highest cumulative number of impetigo consultations during P1. The cumulative number of impetigo consultations in the adjacent region during P1 was marginally higher (64.5) than that of the whole country (56.5) and the control region (56.8).

Table 2 depicts the prescriptions of mupirocin and fusidic acid in the outbreak region and adjacent region. The data suggest that mupirocin prescriptions in both the outbreak and adjacent regions increased during P1 compared with P0. In the outbreak region, fusidic acid was prescribed more frequently in P1 compared with P0 and P2. In the adjacent region, fusidic acid prescriptions were decreased in P1 compared with P0, and remained similar in P2.

**Table 2:** Number of fusidic acid and mupirocin ointment prescriptions from pharmacies in the outbreak region (n = 2) and adjacent region (n = 2) in the Netherlands per period, 2018–2019

		Jul–Oct 2018 (P0)	Jul–Oct 2019 (P1)	Oct–Dec 2019 (P2)
**Outbreak region**	Fusidic acid	211	239	171
	Mupirocin	13	109	51
**Adjacent region**	Fusidic acid	349	241	239
	Mupirocin	27	76	67

P0: the same calendar period as the peak outbreak period in the year before the peak outbreak period; P1: peak outbreak period; P2: the 3 months after the peak outbreak period.

Mupirocin data include both skin and nose ointment, fusidic acid data include only skin ointment.

Data source: electronic records from pharmacies.

### Microbiological and genomic analysis

Using the EUCAST clinical breakpoints and epidemiological cut-offs (ECOFF), antimicrobial susceptibility testing results showed that the isolates were resistant to fusidic acid (> 1 mg/L), beta-lactams, erythromycin (> 2 mg/L), clindamycin (> 0.25 mg/L) and co-trimoxazole (> 4 mg/L). They were susceptible for mupirocin (ECOFF; 1 mg/L), ciprofloxacin (≤ 1 mg/L; susceptible for increased dosing), doxycycline (≤ 1 mg/L), rifampicin (≤ 0.06 mg/L) and linezolid (≤ 4 mg/L).

**Figure 2:**
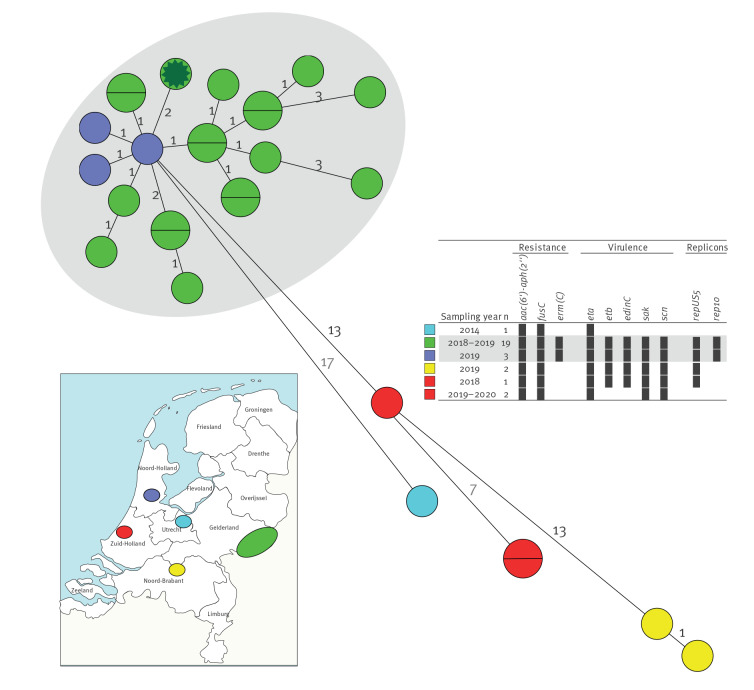
Minimum spanning tree of whole-genome multilocus sequence typing results of 28 meticillin-resistant *Staphylococcus aureus* (MRSA) multilocus variable-number tandem repeat analysis type MT4627 isolates from the Netherlands, January 2008 to January 2020

From the start of MLVA-typing by the national MRSA surveillance in January 2008, until January 2020, 48,737 MRSA isolates were MLVA-typed in the national MRSA surveillance. In total, 58 isolates were MLVA-type MT4627, including 57 from the outbreak and one from 2014. Of these, 28 MT4627 isolates were sequenced, including the 2014 isolate, which was included for comparison. Thirty outbreak region isolates were not sequenced because of the expected high similarity with other outbreak region isolates. All isolates were *mecA*-positive with SCC*mec* type V and belonged to the classical MLST ST 121. Figure 2 shows the minimum spanning tree based on the wgMLST results. Of 28 isolates, 22 were highly related with an allelic difference of ≤ 10 and with similar virulence and antimicrobial resistance genes and plasmid replicons. Nineteen were from the outbreak region (in green) and three from the epidemiologically linked north-western cases (in purple). Six isolates from other regions with no epidemiological link were also related but differed slightly with allelic differences of at least 13, comprising changes of *erm(C)*, some virulence factors and some replicons. Among these were also the isolates from 2014 and 2018 that were not from the outbreak region. Comparison to international genomes showed a difference of 482 alleles between Dutch isolates and the nearest isolate in wgMLST (Supplementary Figure S1). In the comparison, we used 461 NCBI entries comprising all complete *S. aureus* chromosomes in the NCBI database on 8 October 2019 and four read sets from the SRA database [18]. Approximately 55% of the NCBI set were MRSA and no isolate was closely related to the outbreak strain.

*eta*: exfoliative toxine gene A, *etb*: exfoliative toxine gene B, *edinC*: epidermal cell differentiation inhibitor, *fusc*: fusidic acid resistance gene, *erm(C)*: macrolides, lincosamides and streptomycin B resistance gene.

Circles represent the isolates, which are connected to the closest relative. Larger circles include two identical isolates based on wgMLST. The colours represent the different regions with MRSA MT4627 isolates as depicted in the geographical map. Green circles represent isolates from the outbreak region. The circle with the star includes the isolate from the potential primary case from 2018. The isolate from 2014 was not part of the case definition, but was included for comparison with outbreak isolates. The depicted numbers between the isolates represent the allelic distance. A cluster is indicated by a grey halo and is defined as ≥ 2 isolates with an allelic difference of ≤ 15 and with similar virulence and antimicrobial resistance genes and plasmid replicons. The table includes the most important detected virulence factors, antimicrobial resistance genes and plasmid replicons. The map of the Netherlands shows the place of residence of the cases.

Data source: Dutch MRSA surveillance database in BioNumerics.

AMR genes of all sequenced isolates are shown in Supplementary Table S3. All isolates contained resistance genes potentially conferring resistance to beta-lactam antibiotics, trimethoprim, aminoglycosides and fusidic acid, and all but seven isolates contained the *erm(C)* gene conferring resistance to macrolides, lincosamides and streptogramin B. There were no genes or known mutations found in the *folP* gene (encoding for dihydropteroate synthase), encoding sulfamethoxazole resistance [19].

Potential virulence factors are shown in Supplementary Table S4. All isolates were negative for genes encoding PVL and toxic shock syndrome toxin TSST-1. Cluster isolates contained the chromosomally localised fusidic acid resistance gene *fusC* [20] and the exfoliative toxin A (*eta*) gene. We assume that *eta* is chromosomally localised since *eta* was located on contigs that were larger than previously found *S. aureus* plasmids and it is known to be located on a bacteriophage [21]. The cluster isolates contained at least two plasmids, one containing the *erm(C)* gene and rep10 plasmid replicon and one containing exfoliative toxin B (*etb*) gene, epidermal cell differentiation inhibitor C (*edinC*) and replicon repUS5 (Figure 2). Previous reports described plasmids with *erm(C)* of a similar size [22], and *etb* and *edinC* were on the same contig in all isolates [23]. The combination of *eta*, *etb* and *edinC* was not present in 6,265 sequenced non-MT4627 isolates in our sequenced MRSA collection. Several other more common virulence factors were found: enterotoxins, haemolysins, leukocidins (other than PVL), aureolysin, staphylokinase, staphylococcal complement inhibitor and serin proteases.

## Outbreak control measures

When two children with MRSA were found in the outbreak region in July 2019, the GP contacted the regional clinical microbiologist and municipal health service. The municipal health service contacted the school that the children attended. The clinical microbiologist requested GPs in the outbreak region to submit samples for culture in case of impetigo and advised GPs to ensure adequate general hygiene measures. Impetigo was still primarily treated with fusidic acid. However, in case of a positive MRSA culture, impetigo cases were mostly treated with mupirocin ointment on the lesions and in the nose and betadine scrub until lesions were dissolved with a maximum of 14 days. The municipal health service monitored the outbreak and was notified in case of MRSA cultures in the outbreak region. They informed GPs in the outbreak region on the course of the outbreak. Contact investigations were undertaken and family members of impetigo cases were requested to use a daily betadine scrub until lesions of impetigo cases were dissolved. When the outbreak progressed, children with impetigo were not allowed to go to school or day care centres. One school/day care centre had 18 positive children between June and August 2019, for which the regional municipal health service provided support with similar advice as above. The late recognition of the school outbreak was caused by the initial delay in microbiological culture information. The RIVM used the Epidemic Intelligence Information System platform provided by European Centre for Disease Prevention and Control (ECDC) to report this outbreak, but no countries reported similar cases. Collaboration between GPs, clinical microbiologists, the national public health institute and the regional municipal health service led to a rapid decrease in the number of new cases after 3 months.

## Discussion

An MRSA strain belonging to ST 121 with the rare MLVA-type MT4627 caused a community outbreak of impetigo in the eastern part of the Netherlands. The cases were mainly young children and only 10 of the 57 cases were adults. The strain isolates were negative for genes encoding PVL and toxic shock syndrome toxin TSST-1, but contained *eta* and *etb* genes encoding exfoliative toxins, and virulence factor *edinC*. The outbreak strain was resistant to beta-lactam antibiotics, fusidic acid, erythromycin, clindamycin and co-trimoxazole. A collaboration between GPs, clinical microbiologists, the national public health institute and the regional municipal health service led to recognition of the outbreak and development of several interventions, resulting in a rapid decrease in the number of new cases after 3 months.

The clinical presentation was impetigo with lesions of the face, arms, legs, buttocks, and occasionally, but rarely, of the chest and abdomen. An atypical clinical presentation of impetigo was noticed by one GP in the outbreak region and five GPs from the adjacent region and more patients with bullous impetigo were reported. Severe disease was found in only one impetigo case who was admitted to the hospital with generalised bullous impetigo. Interestingly, the MRSA isolates of this outbreak contained exfoliative toxin genes which are associated with SSSS and bullous impetigo [7]. Exfoliative toxins bind to a keratinocyte adhesion protein and cause epidermal dissociation of the human epidermis. The outbreak strain also contained the *edinC* gene, affecting the actin cytoskeleton in the host cell and thereby promoting bacterial colonisation and host tissue invasion [7].

The outbreak was localised in five municipalities in the eastern part of the Netherlands, but data from the GP questionnaires and mupirocin prescriptions suggested that the outbreak was also present in the adjacent region. It is unfortunate that several impetigo cases and family members were not cultured. These findings indicate that the real outbreak extent is difficult to estimate.

The skin infections did not respond to topical fusidic acid and orally administered antibiotics. Mupirocin was the remaining agent of choice which is usually reserved for MRSA carriers and meticillin-susceptible *S. aureus* (MSSA) patients with recurrent skin infections [8]. Resistance to mupirocin was not found, but has been described in CO-MRSA and CO-MSSA, including ST 121 [6,24].

CO-MRSA strains are more virulent and transmissible than traditional healthcare-onset MRSA strains [1,4]. Interventions to prevent further spread of MRSA in the community are different from healthcare-onset MRSA. Numerous studies have evaluated MRSA transmission within hospitals, but the impact of interventions on MRSA transmission dynamics within schools and households is limited. Data from a recent study by *Mork et al.* [25] demonstrated that MRSA introductions frequently occurred via children. During the present outbreak, spread in families was found in 15 of 41 cases, and one school/day care centre encountered an outbreak among 18 children. General infection prevention measures were recommended since a specific protocol for MRSA outbreaks in the community is missing. The municipal health service coordinated all activities.

In general, *S. aureus* ST 121 strains are considered as more virulent strains due to the production of PVL in more than 90% of cases, in addition to the presence of enterotoxins, haemolysins, leukocidins and exfoliative toxins [26]. Approximately 90% of *S. aureus* ST 121 strains are MSSA and they have spread globally, including to European countries such as France, Germany, Portugal and Spain [26]. Interestingly, PVL-positive MSSA ST 121 has also been found in rabbit farming, associated with the most chronic staphylococcal rabbit infections in commercial rabbitries in China [27]. The MRSA strain ST 121 detected in this outbreak is very rare. Reports on CO-MRSA strains belonging to ST 121 that are fusidic acid-resistant and that carry exfoliative toxin genes have not been found. Using our wgMLST data and those of downloaded NCBI and SRA sequences, allelic differences of more than 400 with other ST 121 strains were found.

The origin of the outbreak is unclear. The first case with MLVA-type MT4627 in 2018 had not recently travelled abroad, whereas the first case in 2019 had travelled to Germany. There are no reports on this specific MRSA strain from Germany and MRSA prevalence in Germany is relatively low, although higher than in the Netherlands [9,28]. There were no known links to animal contact or previous foreign hospital admissions among cases.

Our study has some limitations. First, the study is descriptive without a case control or a case–case or cohort study design. Second, we were limited in collection of clinical and epidemiological data and could not determine the precise spread of this strain among family members, relatives and school/day care centres. The most important limitation we encountered is the lack of routine culturing of impetigo lesions.

Up to 2.5 years after the outbreak period, only one more case with the outbreak MLVA-type occurred in the outbreak region and two in the adjacent region. However, in the south-western part of the country, new cases with impetigo are being found since the end of 2020, mostly in children and small family clusters. This indicates that spread has stopped in the outbreak region, but is continuing at a slow pace in another region.

This CO-MRSA outbreak in the Netherlands highlights the risk of strains appearing with a combination of drug-resistance and threatening virulence factors, and that this can occur even in countries with low prevalence of antimicrobial resistance. Intensive MRSA surveillance and collaboration between multiple disciplines are of great importance in early recognition and control of community-onset MRSA outbreaks.

## Supplementary Data

33000 Supplementary Tables

## Supplementary Data

225000 Supplementary Figure
