# Supplementary material for: An unusual outbreak in the Netherlands: community-onset impetigo caused by a meticillin-resistant Staphylococcus aureus with additional resistance to fusidic acid, June 2018 to January 2020
Source: Euro Surveill. 2022 Dec 8;27(49):2200245. doi: 10.2807/1560-7917.ES.2022.27.49.2200245 (PMC9732922; doi:10.2807/1560-7917.ES.2022.27.49.2200245)
Supplement: Supplementary Figure [file 22-00245_VENDRIK_Supplementary_Figure.pdf]

This supplementary material is hosted by *Eurosurveillance* as supporting information alongside the article 'An unusual outbreak in the Netherlands: community-onset impetigo caused by a meticillin-resistant *Staphylococcus aureus* with additional resistance to fusidic acid, June 2018 to January 2020', on behalf of the authors, who remain responsible for the accuracy and appropriateness of the content. The same standards for ethics, copyright, attributions and permissions as for the article apply. Supplements are not edited by *Eurosurveillance* and the journal is not responsible for the maintenance of any links or email addresses provided therein.

Supplementary Figure S1. Whole-genome multi-locus sequence typing (wgMLST)-based minimum spanning tree with 461 NCBI entries and four SRA read sets of *S. aureus* and 22 CC121 methicillin-resistant *S. aureus* (MRSA) outbreak isolates from the Netherlands.

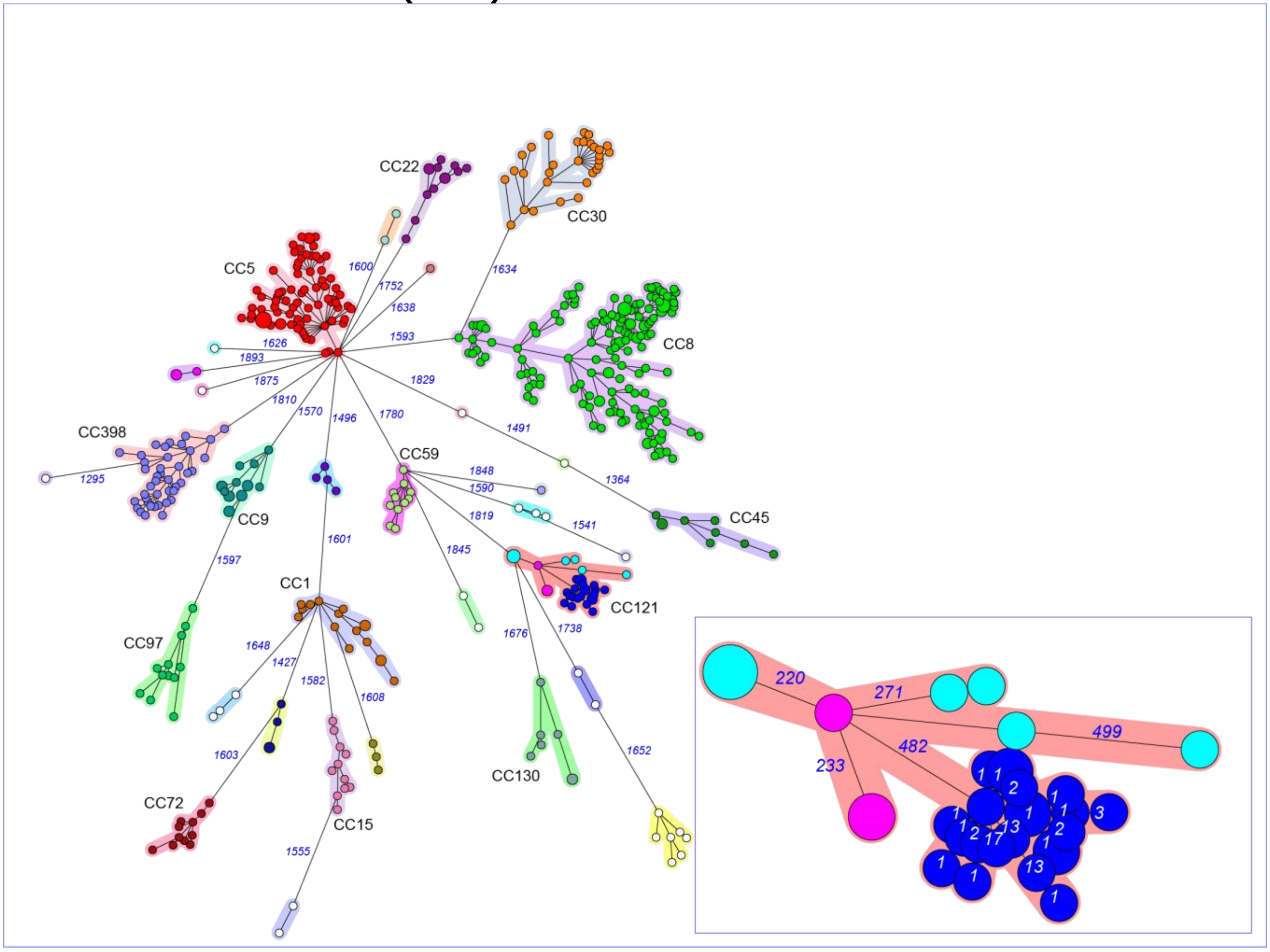

Classical MLST clonal complexes have distinct colours and wgMLST allelic distances between the clonal complexes (CCs) are indicated by blue numbers in italics. The inset shows an enlarged view of the 10 CC121 isolates in the NCBI database and the 22 outbreak isolates from the Netherlands. The dark blue circles are the MRSA outbreak isolates from the Netherlands, light blue circles are NCBI methicillin-susceptible *S. aureus* (MSSA) strains, and the hot pink circles are NCBI MSSA strains carrying the *eta* gene. The numbers in the inset denote the allelic distances between isolates. Abbreviations: NCBI: National Center for Biotechnology Information, SRA: Sequence Read Archive.
